# Biological Activities of Snowdrop (Galanthus spp., Family Amaryllidaceae)

**DOI:** 10.3389/fphar.2020.552453

**Published:** 2021-02-19

**Authors:** Chee Kei Kong, Liang Ee Low, Wei Sheng Siew, Wei-Hsum Yap, Kooi-Yeong Khaw, Long Chiau Ming, Andrei Mocan, Bey-Hing Goh, Poh Hui Goh

**Affiliations:** ^1^ Biofunctional Molecule Exploratory (BMEX) Research Group, School of Pharmacy, Monash University Malaysia, Subang Jaya, Malaysia; ^2^ Department of Primary Care Medicine, Faculty of Medicine, University of Malaya, Kuala Lumpur, Malaysia; ^3^ Institute of Pharmaceutics, College of Pharmaceutical Sciences, Zhejiang University, Hangzhou, China; ^4^ Key Laboratory of Biomedical Engineering of the Ministry of Education, College of Biomedical Engineering and Instrument Science, Zhejiang University, Hangzhou, China; ^5^ School of Biosciences, Taylor’s University, Subang Jaya, Malaysia; ^6^ PAPRSB Institute of Health Sciences, Universiti Brunei Darussalam, Gadong, Brunei; ^7^ Department of Pharmaceutical Botany, Iuliu Hațieganu University of Medicine and Pharmacy, Cluj-Napoca, Romania; ^8^ Laboratory of Chromatography, Institute of Advanced Horticulture Research of Transylvania, University of Agricultural Science and Veterinary Medicine, Cluj-Napoca, Romania; ^9^ College of Pharmaceutical Sciences, Zhejiang University, Hangzhou, China; ^10^ Health and Well-Being Cluster, Global Asia in the 21st Century (GA21) Platform, Monash University Malaysia, Subang Jaya, Malaysia

**Keywords:** snowdrop, galanthus, bioactivities, galanthamine, lycorine

## Abstract

Snowdrop is an iconic early spring flowering plant of the genus *Galanthus* (Amaryllidaceae). *Galanthus* species (*Galanthus* spp.) are economically important plants as ornaments. Galanthus spp has gained significance scientific and commercial interest due to the discovery of Galanthamine as symptomatic treatment drug for Alzhiermer disease. This review aims to discuss the bioactivities of *Galanthus* spp including anticholinesterase, antimicrobial, antioxidant and anticancer potential of the extracts and chemical constituents of *Galanthus* spp. This review highlights that *Galanthus* spp. as the exciting sources for drug discovery and nutraceutical development.

## Introduction

**FIGURE 1:**
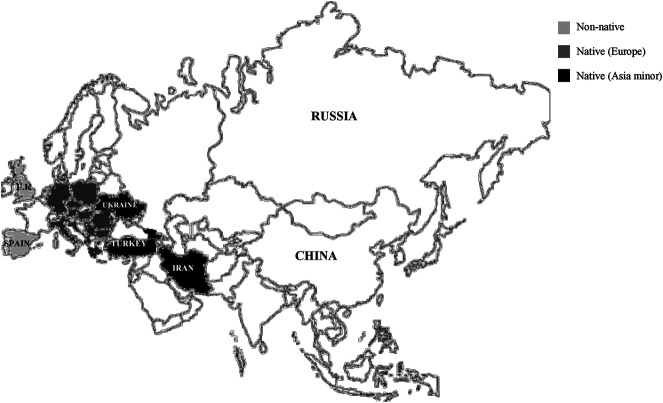
Worldwide’s distribution of the *Galanthus* spp. throughout the United Kingdom and Spain (non-native), Europe (Romania, Bulgaria, etc..) and Southwest Asia (Turkey, Ukraine, Iran).

Amaryllidaceae family comprises about 85 genera and classified into 1,100 perennial bulb species (Bulduk and Karafakıoğlu, 2019). The genus *Galanthus*, commonly known as “snowdrop” belongs to the family of Amaryllidaceae. It is a small genus comprises about 20 species of bulbous perennial herbaceous plants, and a small number of subspecies, varieties and natural hybrids (Rønsted et al., 2013; World Checklist of Selected Plant Families, 2020). *Galanthus* in Greek means “gala” for milk and “anthos” for flower, literally milk-white flowers (Lee, 1999). Native to Europe, their distribution also spread to Asia Minor (southwest Asia) and the Near East, including the eastern parts of Turkey, the Caucasus Mountain and Iran (Figure 1) (Semerdjieva et al., 2019).

Snowdrop are economically important thanks to their ornamental potential and their use as landscape plants (Semerdjieva et al., 2019). Despite their ornamental properties, snowdrops have been used in folk medicine to treat pain, migraine and headache. It contains a variety of secondary metabolites such as flavonoids, phenolics, terpenoids and some important alkaloids that have shown to possess a broad spectrum of biological activities (Semerdjieva et al., 2019). Over the past three decades, many alkaloids isolated from the *Galanthus* spp. including isoquinoline-like compounds such as caranine, narciclasine, tazettine, narwedine and montanine were reported to exhibit acetylcholinesterase inhibitory potential, antibacterial, antifungal, antiparasitic (malaria), antiviral, antioxidant, anticancer, anti-inflammatory activities. (Elgorashi et al., 2003; Orhan and Şener, 2003; Ločárek et al., 2015; Resetár et al., 2017). The main constituents with pharmacological action present in the snowdrop, especially in the bulbs are galanthamine and lycorine (Ayaz et al., 2019). Galanthamine, an alkaloid of *Galanthus woronowii* Losinsk was reported by Proskurnina and Areshknina in 1947, (Proskurnina and Areshknina, 1953). Also, from the same family, galanthamine was purified and characterized from the bulbs of the *G. nivalis* L. by Dimatar PaskovGalanthamine has been used as the promising drug (known as Nivalin) for the symptometric treatment Alzheimer’s disease (AD) (Paskov, 1959; Ayaz et al., 2019). In addition, lectins agglutinin (GNA) were discovered from *Galanthus nivalis*.

In this review, we discuss the traditional uses and report all published data in relation to their secondary metabolites and biological activities of snowdrops.

## The Snowdrop Plants (*Galanthus* spp.)

**FIGURE 2:**
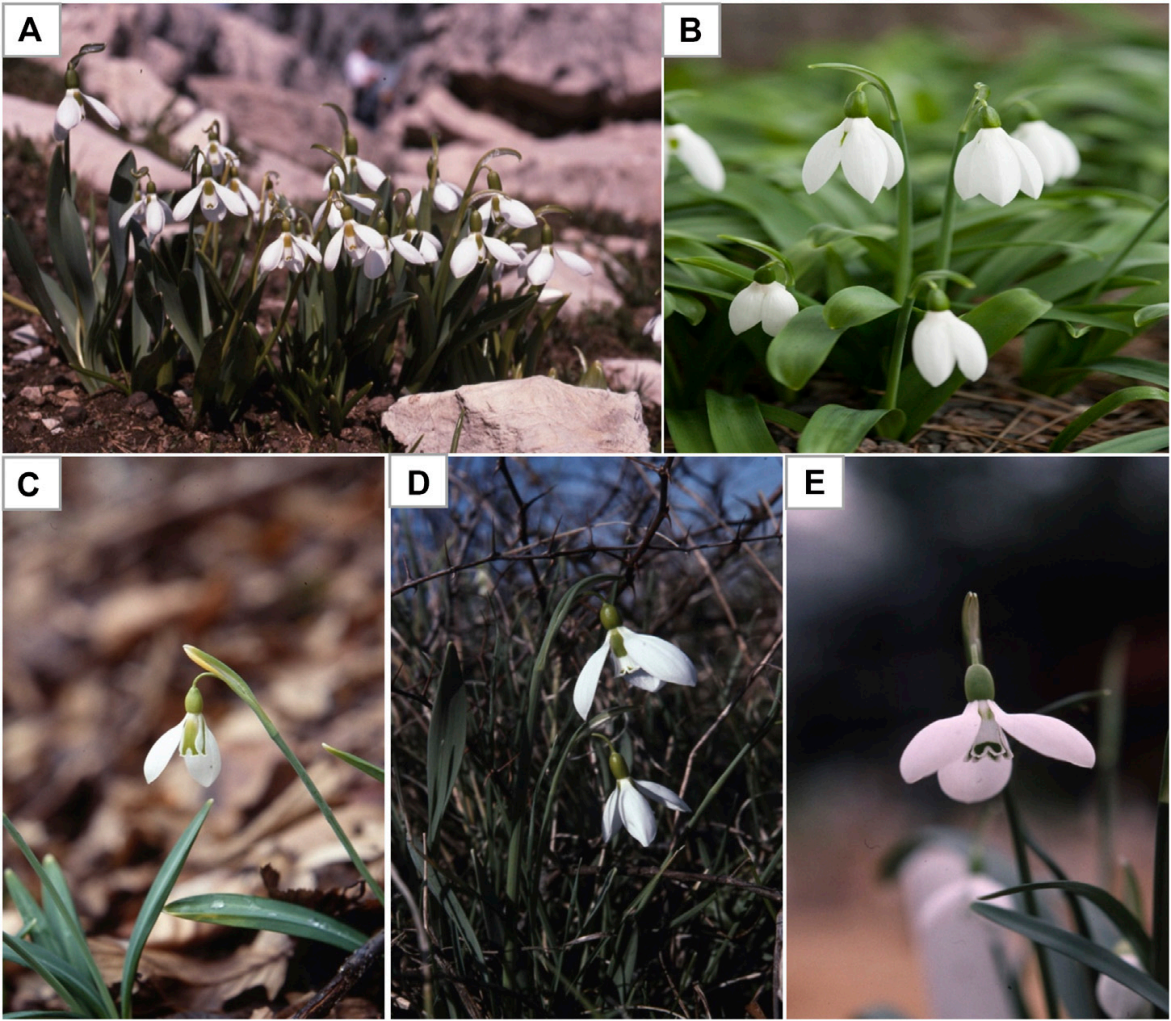
Examples of some commonly found *Galanthus* spp. **(A)** *Galanthus nivalis* **(B)** *Galanthus elwesii* (Giant or great snowdrops) **(B)** *Galanthus gracilis* **(C)** *Galanthus ikariae* **(D)** *Galanthus trojanus.* Adapted from Davis (2011).

Snowdrops are tiny plants (3 to 6 inches tall) with (1 inch or less) white flowers. Each snowdrop bulb produces two linear narrow grassy leaves and a single flower with a delicate small white drooping bell shaped flower. The snowdrop has no petal, but tepal. The outer three are longer pure white, while the smaller inner three are shorter and blushed with green markings (Aschan and Pfanz, 2006). There are many different varieties and species of snowdrop flowers that differs in terms of the size of the tepals and the green markings. As the name suggests, snowdrops are winter-to-spring flowering plants, of which *Galanthus nivalis* is the first and most common species of the genus (Figure 2; Table 1) to bloom during the end of the winter taking advantage of the lack of tree canopy to capture sunlight for photosynthesis and growth (Orhan and Şener, 2003). Wild snowdrops grow in damp soil in the temperate deciduous woodlands, for example oak (*Quercus* spp.), maple (*Acer* spp.), pines (*Pinus* spp.), cedar of Lebanon (*Cedrus libani*), particularly nearby shady areas, near river or streams (Elgorashi et al., 2003). *Galanthus* spp. are difficult to distinguish and classify due to high variability of morphological characteristics which is not clearly definable, which led to multiple taxonomic revisions *Galanthus* over the years (Rønsted et al., 2013). Currently, all species of *Galanthus* are classified as Critically Endangered (CR) under International Union for Conservation of Nature (IUCN) Red List Categories and Appendix II of the Convention on International Trade (CITES) in the list of Wild Fauna and Flora. The endangered status of *Galanthus* is due to its susceptibility to climate change, plucking and forestry and unregulated *Galanthus* bulb trade (International Union for Conservation of Nature, 2018). It is noteworthy that under CITES regulations, only rural communities in many countries are allowed in limited wild harvest and trade of just three species (*G. nivalis, G. elwesii, and G. woronowii*) (Bishop et al., 2001).

**TABLE 1:** *Galanthus* spp.’s common names and scientific names.

Plant common name	Plant full scientific name Kew MPNS	Voucher specimen deposition
Common snowdrop	*Galanthus nivalis* L.	Royal Botanic Gardens, Kew
Giant or great snowdrop	*Galanthus elwesii* Hook.f.	Royal Botanic Gardens, Kew
Graceful or slender snowdrop	*Galanthus gracilis* Celak.	Royal Botanic Gardens, Kew
Ikaria snowdrop	*Galanthus ikariae* Baker.	Royal Botanic Gardens, Kew
Trojanus snowdrop	*Galanthus trojanus* A.P.Davis & Özhatay	Royal Botanic Gardens, Kew
Queen Olga’s snowdrop	*Galanthus reginae-olgae* Orph.	Royal Botanic Gardens, Kew
Subspecies of Queen Olga’s snowdrop	*Galanthus reginae-olgae* Orph. subsp*. vernalis* Kamari	—
Hybrids of *G. nivalis* and *G. plicatus* subsp. *byzantinus*	*Galanthus xvalentinei* nothosubsp. *subplicatus* ^a^	—
Short snowdrop	*Galanthus rizehensis* Stern	Royal Botanic Gardens, Kew
Snowdrop Cilician	*Galanthus cilicicus* Baker.	Royal Botanic Gardens, Kew
Gol-e-Barfi	*Galanthus transcaucasicus* Fomin	—
Pleated snowdrop	*Galanthus plicatus* M.Bieb.	Royal Botanic Gardens, Kew
Subspecies of Pleated snowdrop	*Galanthus plicatus* subsp. *byzantinus* (Baker) D.A.Webb	Royal Botanic Gardens, Kew
Lagodekhsky snowdrop	*Galanthus lagodechianus* Kem-Nath.	—
Green snowdrop or Woronow’s snowdrop	*Galanthus woronowii* Losinsk.	Royal Botanic Gardens, Kew
Krasnov snowdrop	*Galanthus krasnovii* Khokhr.	Royal Botanic Gardens, Kew
˗	*Galanthus alpinus* Sosn.	—
Broad-leaved snowdrop	*Galanthus platyphyllus* Traub & Moldenke (previously known as *G.latifolius*)	—
Caucasian snowdrop	*Galanthus caucasicus* (Baker) Grossh. (now accepted as *Galanthus alpinus* var. *alpinus*)	Royal Botanic Gardens, Kew
Kemularia	*Galanthus kemulariae* Kuth. (now accepted as *Galanthus lagodechianus* Kem-Nath.)	—
Rare snowdrop	*Galanthus shaoricus* Kem-Nath^a^	—
—	*Galanthus peshmenii* A.P.Davis & C.D.Brickell	—

^a^ Not found in http://powo.science.kew.org.

## Snowdrop in Folklore

For centuries, the snowdrops have been used as a remedial herb to ease migraines and headaches. Plaitakis and Duvoisin believed the oldest record on snowdrop (*Galanthus nivalis* L.) was found in ancient Homer’s epic poem, where snowdrop is described as ‘moly’ and used by Odysseus as an antidote against Circe’s poisonous drugs (Plaitakis and Duvoisin, 1983). According to an unconfirmed report in the early 1950s, a Bulgarian pharmacologist noticed people of the remote areas rubbing their foreheads with the plant leaves and bulbs as a folk remedy to relieve nerve pain (Mashkovsky and Kruglikova–Lvova, 1951). Besides, some of the earlier publications had left traces that of evidences on the extensive use of snowdrop in Eastern Europe, such as Romania, Ukraine, the Balkan Peninsula, as well as in some Eastern Mediterranean countries (Heinrich, 2010). However, there were no relevant ethnobotanical literatures for confirmation to be located. Russian pharmacologists reported that local villagers at the foot of the Caucasian mountains in Georgia used the decoction of the bulbs of wild snowdrop (*G. woronowii* Los.) for the treatment of poliomyelitis in children (Sidjimova et al., 2003). Besides, an old glossary also classified snowdrop as cardiotonic, stomachic and emmenagogue (Baytop, 1999). The use of *Galanthus* herb has shown to increase the flow of menstrual blood to cure dysmennorhea or oligomennorhea, and was once used to induce an abortion if in the early stages of pregnancy (Baytop, 1999). Although snowdrops have a long traditional use in folk medicines, the chemical constituent recently become a commercial proposition (Ay et al., 2018). Snowdrops have attracted attention due to its pharmacological potential (wild snowdrops trade) and the chemical diversity (Sidjimova et al., 2003). It is interesting to note that, the bulb of the plant contains a chemical called phenanthridine alkaloid, which is toxic to animals including dogs and cats and may lead to gastrointestinal disorders in humans. Lycorine, the phenanthridine alkaloid is used in herbal medicines and pharmaceutical drugs over the years (Lamoral-Theys et al., 2009).

## Biological Substances of Snowdrop and Their Ethnopharmacology

**FIGURE 3:**
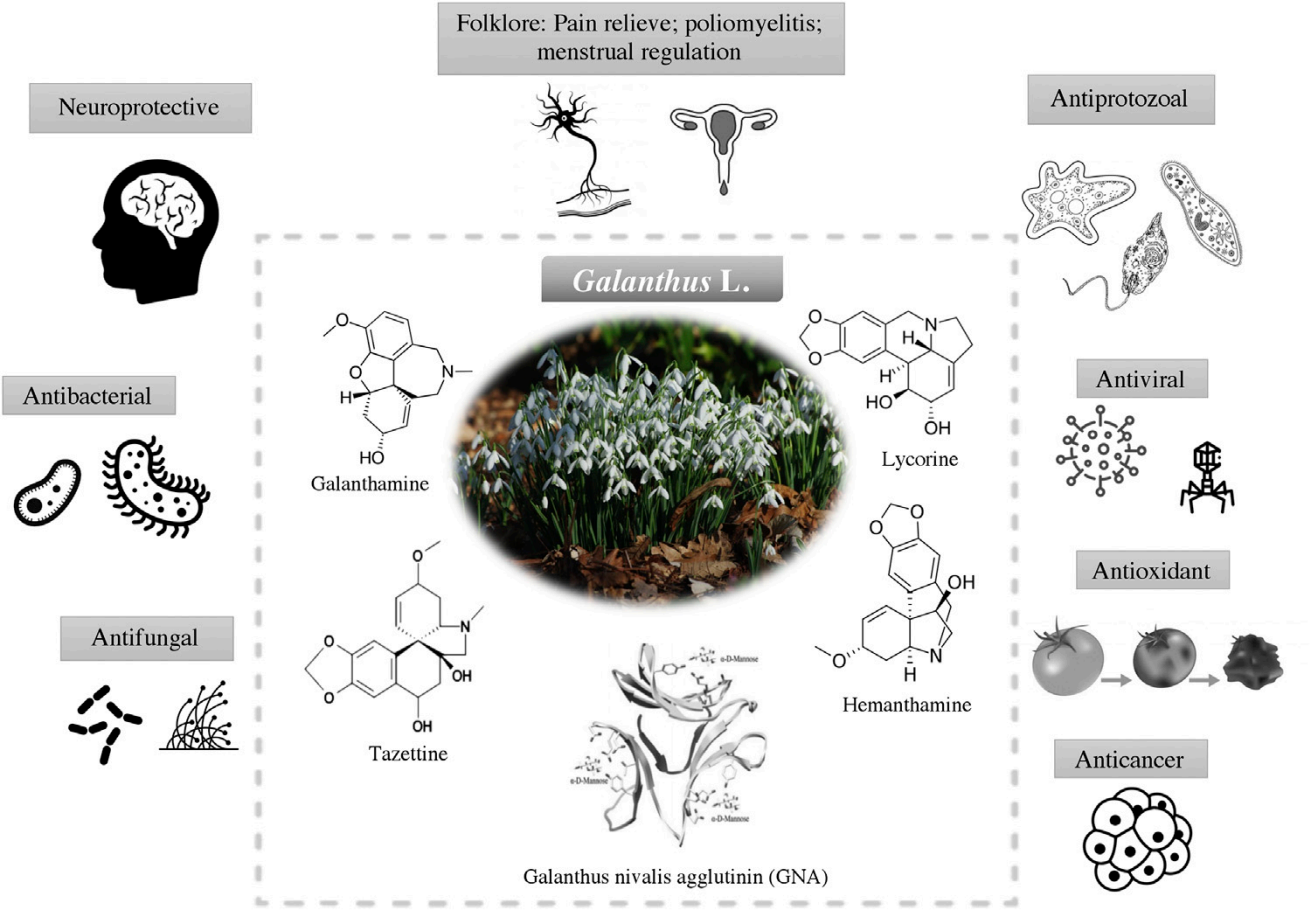
Biological activities of *Galanthus* spp. and their main bioactive constituents (alkaloids and plant lectin) that contributed to these activities.

Having evolved over millions of years and wide application in traditional medicine. The discovery of new drug from snowdrops begin in the new decade. The discovery of galanthamine has attracted the interest from scientific community to further explore the relationships between the underexplored pharmacological properties of snowdrops and its chemical space. This including the antimicrobial, antioxidant and anticancer activities (Figure 3). The active compounds which are responsible for the biological activities are listed in Table 2.

**TABLE 2:** Pharmacological activities of Snowdrop.

Biological activities	Species	Plant parts	Type of extract	Phenotypic activity	Effective dose^a^	Positive control	Possible mechanism of action	Compounds	Isolation/Detection methods	References
Cholinesterase	*Galanthus nivalis* L.	Bulb	Ethanol extract	AChE	96%	—	Inhibit the cholinesterase enzyme from breaking down ACh, increasing both the level and duration of the neurotransmitter action.	—	—	Rhee et al. (2003)
*Galanthus elwesii* Hook.f.	Bulb	Chloroform: methanol (1:1)	AChE	73.18%	Galanthamine		—	Orhan and Şener (2005)	
			AChE	77.23%					
*Galanthus ikariae* Baker	Bulb	Chloroform: methanol (1:1)	AChE		Lycorine	Column chromatography and preparative TLC			
			Chloroform: methanol extract lycorine	75.56%	Tazettine				
			Tazettine	3.16 μM	Galanthamine				
			crinine galanthamine	3.20 μM	Crinine				
				3.20 μM	3-epihydroxybulbispermine				
				3.20 μM	2-demethoxymontanine				
				3.20 μM					
*Galanthus reginae-olgae Orph.* subsp. *vernalis* Kamari			AChE	76.96%				Conforti et al. (2010)	
			Alkaloid extract						
	Aerial	Methanol extract	AChE						
			Methanol extract	15.2 ± 0.81%	Physostigmine		1-metil-4-etossi-,δ, (3)pirrolin-2-one, Neophytadiene, Exadecanoic acid, methyl ester, Exadecanoic acid, 9,12-Octadecandienoic acid, methyl ester,[E,E], 9,12,15-octadecantrienoic acid, methyl ester,[Z,Z,Z], 2-exadecen-1-ol-3,7,11,15-tetramethyl-[R-[R*,R*-(E)]], 9,12,15-octadecantrienal, 9,12-octadecandienoic acid [Z,Z]-2-idrossi-1-[idrossimethyl] ethylester, 2-monolinolenin, 1-octadecene, 9-α-fluoro-5-α-cholest-8(14)-ene-3,15-dione, Vitamin E, Ergost-5-en-3-ol, (3.β.,24 E), Stigmast-5-en-3-ol, (3.β.,24 S), Stigmast-5,24(38)-dien-3-ol, (3.β.,24 E)		GCMS
			Hexane fraction	1.2 ± 0.04%	—		Galanthamine, Lycorine, Tazettine		
			Ethyl acetate fraction	1.2 ± 0.06%					
			Dichloromethane fraction	11.8 ± 0.72%					
	Bulb	Methanol extract	AChE				Neophytadiene, Exadecanoic acid, Methyl ester, 9,12-Octadecandienoic acid, methyl ester,[E,E], 9,12,15-octadecantrienoic acid, methyl ester,[Z,Z,Z], 9,12-octadecandienoic acid [Z,Z]-2-idrossi-1-[idrossimethyl] ethylester Galanthamine, Lycorine, Tazettine, Crinine, Neronine		
			Methanol extract	18.2 ± 0.93%					
			Hexane fraction	7.8 ± 0.49%					
			Ethyl acetate fraction	5.0 ± 0.42%					
			Dichloromethane fraction	38.5 ± 0.49%					
*Galanthus gracilis* Celak.	Bulb	Alkaloid fraction	AChE	IC_50_: 11.82 μg/ml	Galanthamine		8-*O*-demethylhomolycorine, Homolycorine ,Galanthindole . Tazettine Lycorine, Galanthamine	GCMS	Bozkurt-Sarikaya et al. (2014)
			Alkaloid fraction						
	Aerial	Alkaloid fraction	AChE	IC_50_: 25.5 μg/ml			Homolycorine, 8-*O*-demethylhomolycorine, Galanthindole, Tazettine		
			Alkaloid fraction						
*Galanthus xvalentinei* nothosubsp. *subplicatus*	Bulb	Alkaloid fraction	AChE	IC_50_: 21.31 μg/ml			Lycorine ismine		Sarikaya et al. (2013)
			Alkaloid fraction						
	Aerial	Alkaloid fraction	AChE	IC_50_: 16.32 μg/ml			Tazettine, 11-*O*-(3'-Hydroxybutanoyl) hamayne, 3-*O*-(2''-Butenoyl)-11-*O*-(3'-hydroxybutanoyl) hamayne		
			Alkaloid fraction						
*Galanthus woronowii Losinsk*	Aerial and Bulb	Alkaloid extract	AChE		Galanthamine (IC_50_: 0.15 μM)		Galanthine, Galanthamine, 2-O-(3’-hydroxybutanoyl)lycorine, Narwedine, 1-O-acetyl-9-O-methylpseudolycorine, O-methylleucotamine, Sternbergine, Lycorine, Sanguinine, Salsoline	Column Chromatography	Bozkurt et al. (2013a)
			Galanthine Narwedine	IC_50_: 7.75 μM					
			O-methylleucotamine	IC_50_: 11.79 μM					
			Sternbergine Sanguinine	IC_50_: 16.42 μM					
			1-O-acetyl-9-O-methylpseudolycorine	IC_50_: 0.99 μM					
				IC_50_: 0.007 μM					
				IC_50_: 78.7 μM					
*Galanthus rizehensis* Stern	Bulb	—	AChE	IC_50_: 12.94 μg/ml			Lycorine, Tazettine,	GCMS	Bozkurt et al. (2013b)
							Galanthamine		
*Galanthus cilicicus* Baker	Bulb	Alkaloid fraction	AChE	IC_50_: 0.407 μg/ml	Galanthamine AChE IC_50_: 0.043) μg/ml; BuChE 0.711 μg/ML		Galanthamine, Tazettine,	GCMS	Kaya et al. (2017)
			Alkaloid fraction				Galanthindole		
			BuCHE	IC_50_: 8.14 μg/ml					
			Alkaloid fraction						
	Aerial	Alkaloid fraction	AChE	IC_50_: 0.154 μg/ml			Haemanthamine, Tazettine, Galanthindole		
			Alkaloid fraction						
			BuCHE	IC_50_: 82.18 μg/ml					
			Alkaloid fraction						
*Galanthus elwesii* Hook.f.	Aerial (Location: Karaburun, Izmir)	Alkaloid fraction	AChE	IC_50_: 0.72 μg/mL μg/ml	Galanthamine (AChE IC_50_: 0.04) μg/ML, BuCHE IC_50_: 0.711		Hordenine, Anhydrolycorine, Galanthamine, O-methylleucotamine Sanguinine, 11,12-Didehydroanhydrolycorine, Incartine, Oxoincartine	GCMS	Bozkurt et al. (2017)
			Alkaloid fraction						
			BuCHE	IC_50_:6.56 μg/ml					
			Alkaloid fraction						
	Bulb (Location: Karaburun, Izmir)	Alkaloid fraction	AChE	IC_50_: 2.20 μg/ml	Galanthamine (IC_50_: 0.04) μg/ml		Hordenine, Anhydrolycorine, Lycorine, Galanthamine, O-methylleucotamine, Sanguinine, Incartine, Oxoincartine		
			Alkaloid fraction						
			BuCHE	IC_50_: 15.84 μg/ml					
			Alkaloid fraction						
	Aerial (Location: Akseki, Antalya)	Alkaloid fraction	AChE	IC_50_:	Galanthamine (IC_50_: 0.04) μg/ml		Galanthindole, Haemanthamine 6-O-methylpretazettine, Galanthindole, 1-acetyl-Β-Carboline, Pinoresinol		
			Alkaloid fraction	15.72 μg/ml					
	Bulb (Location: Akseki, Antalya)	Alkaloid fraction	AChE	IC_50_: 10.52 μg/ml	Galanthamine (IC_50_: 0.711) μg/ml		Galanthindole, Haemanthamine 6-O-methylpretazettine, Galanthindole, 1-acetyl-Β-Carboline, Pinoresinol		
			Alkaloid fraction						
	Aerial (Location: Demirci, Manisa)	Alkaloid fraction	AChE	IC_50_: 6.25 μg/ml	Galanthamine (IC_50_: 0.04) μg/ml		Galanthamine, Sanguinine, Demethylhomolycorine, *O*-methylleucotamine, Lycorine, Anhydrolycorine, Hordenine, Ismine,, 2,11-didehydro-2-dehydroxylycorine, Assoanin, 11,12-didehydroanhydrolycorine, Hippeastrine		
			Alkaloid fraction						
			BuCHE	IC_50_: 9.52 μg/ml					
			Alkaloid fraction						
	Bulb (Location: Demirci, Manisa)	Alkaloid fraction	AChE	IC_50_: 15.65 μg/ml	Galanthamine (IC_50_: 0.711) μg/ml		Galanthamine, Incartine, Lycorine, Anhydrolycorine And Hordenine, Ismine, Demethylmaritidine, 2,11-Didehydro-2-Dehydroxylycorine, Assoanine, 11,12-Didehydroanhydrolycorine, Hippeastrine		
			Alkaloid fraction						
			BuCHE	IC_50_: 15.85 μg/ml					
			Alkaloid fraction						
*Galanthus peshmenii* A.P.Davis and C.D.Brickell	Whole plant	–	AChE	IC_50_: 49.04 μg/ml	Galanthamine (AChE IC_50_: 0.043) μg/ml (BuCHE IC_50_: 0.711 ml)		Homolycorine, Ismine, Graciline, Galanthindole, Tazettine, Demethylhomolycorine, Galwesine	GCMS	Bozkurt et al. (2020)
			BuCHE	IC_50_: 42.05 μg/ml				
*Galanthus Gracilis* Celak.	Bulb	Alkaloid fraction	AChE	IC_50_: 27.51 μg/ml			*O*-methylnorbelladine, ismine, graciline, 5,6-dihydrobicolorine, vitattine, galanthindole, 11,12-dehydrolycorene, tazettine, 11-OH vittatine, lycorine, homolycorine, pinoresinol	GCMS
			BuCHE	IC_50_: 35.72 μg/ml				
	Aerial	Alkaloid fraction	AChE	IC_50_: 61.05 μg/ml			Graciline, 5,6-dihydrobicolorine, galanthindole, 6-*O*-methylpretazettine, tazettine, homolycorine, demethylhomolycorine, 3-*O*-demethylmacronine, hippeastrine	
			BuCHE	IC_50_: 69.83 μg/ml				
*Galanthus krasnovii* Khokhr.	Bulb	Alkaloid fraction	AChE	IC_50_: 8.26 μg/ml			Hordenine, *O*-methylnorbelladine, 1-acetyl-β-Carboline, Trisphaeridine, 5,6-dihydrobicolorine, Vittatine, 11,12-dehydrolycorene, Demethylmaritidine, Anhydrolycorine, 11-OH vittatine, 11,12-didehydroanhydrolycorine, Pseudolycorine	GCMS
			BuCHE	IC_50_: 6.23 μg/ml				
	Aerial	Alkaloid fraction	AChE	IC_50_: 23.52 μg/mL			Hordenine, *O*-methylnorbelladine, 1-acetyl-B-Carboline, 11,12-dehydrolycorene, Anhydrolycorine, 11-OH vittatine, 11,12-didehydroanhydrolycorine, Pseudolycorine	
			BuCHE	IC_50_:14.91 μg/ml				
**Antibacterial**	*Galanthus transcaucas-icus* Fomin	Bulb	Ethanol extract	Ethanol extract	MIC: 9.275 mg/ml	—	Disruption of membrane structure by inhibiting enzymes in cell wall biosynthesis, protein synthesis and nucleic acid synthesis.		—	Sharifzadeh et al. (2010)
			Chloroform fraction	Chloroform fraction	MIC: 1.17 mg/ml	—			—	
	*Galanthus plicatus* subsp. *byzantinus* (Baker) D.A. Webb	Aerial	Ethanol extract	S.epidermidis: S.pyrogene	Zone of inhibition:	Chloramphenicol: *S. epidermis* 29.75 mm; S. pyogenes			—	Turker and Koyluoglu (2012)
				*S. epidermis*	7.25 mm	33.75 mm, *P. vulgaris*				
				*S. pyogenes*	12.50 mm	20.50 mm; *K. pneumonia*				
				*P. vulgaris*	8.25 mm	28.50 mm				
				*K. pneumonia*	7.25 mm					
	*Galanthus transcaucas-icus* Fomin	Bulb	Methanol extract	*B. subtilis*	0.82 cm	Kanamycin		2-furancarboxaldehyde , Gallic Acid, Syringic Acid, Catecin And Ferulic Acid	HPLC, GCMS	Karimi et al. (2018)
				*B. cereus*	0.71 cm	*B. subtilis*: 1.28;				
				*S.aureus*	0.35 cm	*B. cereus* 1.36; *S. aureus* 1.17;				
				*E. coli*	0.85 cm	*E. coli* 1.42;				
				*P. aeruginosa*	0.46 cm	*P. aeruginosa* 1.21 cm		2,3-butanediol, Acetic acid, Naringin, Quercetin, Apigenin, Genistein		
		Flower	Methanol extract	*B. subtilis*	1.05 cm					
				*B. cereus*	1.22 cm					
				*S.aureus*	0.76 cm 76 cm					
				*E. coli*	1.16 cm					
				*P. aeruginosa*	0.98 cm					
		Shoot	Methanol extract	*B. subtilis*	1.12 cm			Acetic acid, *n-*hexadecenoic acid, 4H-pyran-4-one, Naringin, Quercetin, Apigenin, Genistein		
				*B. cereus*	1.18 cm					
				*S.aureus*	0.92 cm					
				*E. coli*	1.29 cm					
				*P. aeruginosa*	1.06 cm	Gentamicin		Chlorogenic acid, p*-*coumaric acid, Ferulic acid, Isoquercitrin, Quercitrin	HPLC	Benedec et al. (2018)
	*Galanthus nivalis* L.	Aerial	Ethanol extract	*S. enteritidis*	6 mm	*S. enteritidis*: 19;				
				*E coli*	6 mm	*E coli* 18;				
				*L. monocytogenes*	10 mm	*L. monocytogenes* 10;				
				*S. aureus*	18 mm 8 mm	*S. aureus* 22 mm;				
				*C. albicans*	6 mm	Fluconazole:				
				*A. brasillensis*	16 mm	*C. albicans* 25 mm,				
					16 mm	Amphotericin B:				
						*A. brasillensis*: 21 mm				
				*S. enteritidis*	MIC: 625 mm					
				*E coli*	2,500 mm					
				*L. monocytogenes*	312.5 mm					
				*S. aureus*	19.53 mm					
				*C. albicans*	19.53 mm					
				*A. brasillensis*	1,250 mm					
					78.13 mm78.13 mm					
Antifungal	*Galanthus transcaucas-icus* Fomin	Bulb	Ethanol extract	*C. albicans*	MIC: 150 unit/Ml	—		—		Sharifzadeh et al. (2010)
			Chloroform fraction	*S. aureus*	MIC: 1.17 mg/ml					
	*Galanthus elwesii* Hook.f.	Bulb	Ethanol extract	*C. albican*	MIC:1024 ug/mL	—		Galanthamine, Tazettine	GCMS	Ločárek et al. (2015)
				*C. dubliniensis*	1024 ug/mL					
				*C. glabrata*	512 ug/mL					
				*C. dubliniensis*	512 ug/mL					
			Galanthamine	*C. dubliniensi*	MCF512 ug/mL					
				*L. elongiosporus,*	512 ug/mL					
			Tazzetine	*C. dubliniensi*	512 ug/mL					
				*L.elongiosporus*	512 ug/mL					
	*Galanthus nivalis* L.	Aerial	Ethanol extract	*C. albicans*	Zone of inhibition:6 mm	Fluconazole ( *C. albicans* 25 mm), Amphotericin B ( *A. brasiliensis :* 10 mm)		Chlorogenic acid, *p-*coumaric acid, Ferulic acid, Isoquercitrin, Quercitrin	HPLC	Benedec et al. (2018)
				*A. brasiliensis*	16 mm					
				*A. brasiliensis*	MIC: 78.13 μg/ml					
				*C. albicans*	MIC: 1250 μg/ml	—				
Antiprotozoal	*Galanthus trojanus* A.P.Davis and Özhatay	Whole plant	Arolycoricidine	*T. b. rhodesiense*	IC_50_ 5.99 μg/ml	Melarsoprol ( *T b. rhodesiense*	Direct inhibition of the enzyme involved in the fatty acid biosynthesis (FAS) pathway.	1-*O*-acetyldihydromethylpseudolycorine N-oxide, 11-hydroxyvittatine N-oxide, Arolycoricidine , (+)-haemanthamine, (+)-narcidine, *O*-methylnorbelladine, (–)-stylopine, (–)-dihydrolycorine, protopine, (+)-8-*O*-Demethylmaritidine, Nicotinic acid, Tyramine	Column chromatography, preparative TLC	Kaya et al. (2011)
				*P. falciparum*		IC_50_: 0.004 μg/ml), Benznidazole ( *T. cruzi*				
				*T. b. rhodesiense*		IC_50_: 0.36 μg/ml), Chloroquine (*P. falciparum*				
				*T. b. rhodesiense*	IC_50_ 4.44 μg/ml	IC_50_: 0.0065 μg/ml)				
			(+)-haemanthamine	*T. cruzi*	IC_50_ 3.35 μg/ml					
				*P. falciparum*	IC_50_ 4.44 μg/ml					
				*T. b. rhodesiense*	IC_50_ 1.80 μg/ml					
				*P. falciparum*	IC_50_ 2.75 μg/ml					
			Dihydrolycorine	Cytotoxicity	IC_50_ 3.10 μg/ml					
				L6 cells	IC_50_ 0.23 μg/ml					
				KB cells	IC_50_ > 90 μg/ml					
			Stylopine	*T. b. rhodesiense*	IC_50_ > 50 μg/ml					
				*P. falciparum*	IC_50_ 8.71 μg/ml					
			Protopine	Cytotoxicity	IC_50_ 0.50 μg/ml					
				L6 cells	IC_50_ 63.30 μg/ml					
				KB cells	IC_50_ > 50 μg/ml					
Antiviral	*Galanthus. elwesii* Hook.f.	Bulb	Ethanol extract	Herpes simplex virus	Antiviral conc 8 μg/ml	—	Inhibition of the viral replication and host cell lysis.	Galanthus *nivalis* agglutinin (GNA)		Hudson et al. (2000)
		Bulb	Ethanol extract	Sindbis virus	Antiviral conc 16 μg/ml	—	Direct inactivation of the viral particles.			
	*Galanthus reginae-olgae Orph.* subsp. *vernalis* Kamari	Aerial	Methanol extract	DPPH	IC_50_: 39 ± 0.067 μg/ml	DPPH:Ascorbic acid (2 ± 0.011 μg/ml)	Direct inhibition of ROS	—	GCMS	Conforti et al. (2010)
				Lipid Peroxidation	1000 μg/ml	Lipid Peroxidation: Propyl gallate (7 ± 0.017 μg/ml)	Inhibition of formation of free malonaldehyde (MDA) as the result of oxidation in lipid			
				β-Carotene bleaching	11 ± 0.016 μg/ml	β-Carotene bleaching: Propyl gallate; (1 ± 0.009 μg/ml), DPPH:Ascorbic acid (2 ± 0.011 μg/ml)	Inhibition of peroxyl radicals damage on β-Carotene			
			Hexane fraction	DPPH	IC_50_: > 1,000 μg/ml			1-metil-4-etossi-, δ, (3)pirrolin-2-one, Neophytadiene, Exadecanoic acid, methyl ester, Exadecanoic acid, 9,12-Octadecandienoic acid, methyl ester, [E, E], 9,12,15-octadecantrienoic acid, methyl ester, [Z, Z,Z], 2-exadecen-1-ol-3,7,11,15-tetramethyl-[R-[R*,R*-(E)]], 9,12,15-octadecantrienal, 9,12-octadecandienoic acid [Z,Z]-2-idrossi-1-[idrossimethyl] ethylester, 2-monolinolenin, 1-octadecene, 9-α-fluoro-5-α-cholest-8(14)-ene-3,15-dione, Vitamin E, Ergost-5-en-3-ol, (3.β.,24 E), Stigmast-5-en-3-ol, (3.β.,24 S), Stigmast-5,24(38)-dien-3-ol, (3.β.,24 E)		
				Lipid Peroxidation	>1000 μg/ml					
				β-Carotene bleaching	16 ± 0.045 μg/ml					
			Alkaloid fraction	DPPH	IC_50_: 146 ± 0.238 μg/ml			Galanthamine, Lycorine, Tazettine		
				Lipid Peroxidation	74 ± 0.139 μg/ml					
				β-Carotene bleachingβ-Carotene bleaching:	9 ± 0.018 μg/ml					
			Ethyl acetate fraction	DPPH	IC_50_: 10 ± 0.020 μg/ml					
				Lipid Peroxidation	962 ± 1.231 μg/ml					
				β-Carotene bleaching	12 ± 0.017 μg/ml					
		Bulb	Methanol extract	DPPH	IC_50_: 29 ± 0.051 μg/ml	—		Neophytadiene, Exadecanoic acid, methyl ester, 9,12-Octadecandienoic acid, methyl ester,[E,E], 9,12,15-octadecantrienoic acid, methyl ester,[Z,Z,Z], 9,12-octadecandienoic acid [Z,Z]-2-idrossi-1-[idrossimethyl] ethylester		
				Lipid Peroxidation	1000 μg/ml					
				β-Carotene bleaching	92 ± 0.231 μg/ml					
			Hexane fraction	DPPH	IC_50_: > 1,000 μg/ml					
				Lipid Peroxidation	1,000 μg/ml					
				Lipid Peroxidation	>100 μg/ml					
				β-Carotene bleaching						
			Alkaloid fraction	DPPH	IC_50_: 15 ± 0.031 μg/ml			Galanthamine, Lycorine, Tazettine, Crinine, Neronine		
				Lipid Peroxidation	273 ± 0.345 μg/ml					
				β-Carotene bleaching:	15 ± 0.035 μg/ml					
			Ethyl acetate fraction	DPPH	IC_50_: 148 ± 0.231 μg/ml	—				
				Lipid Peroxidation	1000 μg/ml					
				β-Carotene bleaching:	10 ± 0.019 μg/ml					
	*Galanthus transcaucas-icus* Fomin	Bulb	Methanol extract	DPPH	IC_50_: 171.07 μg/ml	Vitamin C (65.62 μg/ml), Vitamin E	Direct inhibition of ROS.	2-furancarboxaldehyde	HPLC, GCMS	Karimi et al. (2018)
						(60.39 μg/ml), BHT (83.75 μg/ml)				
		Flower		DPPH	IC_50_: 132.61 μg/ml			2,3-butanediol, Acetic acid		
		Shoot		DPPH	IC_50_: 125.07 μg/ml			Acetic acid, *n-*hexadecenoic acid, 4H-pyran-4-one		
	*Galanthus transcaucas-icus* Fomin	Bulb		ABTS	IC_50_: 292.73 ± 1.94 μg/ml	Trolox (191.36 ± 2.02 μg/ml)		Gallic acid, Syringic acid, Catechin, Ferulic acid, Naringin, fla, rutin		
		Flower		ABTS	IC_50_: 267.47 ± 1.45 μg/ml			Gallic acid, Resorcinol, Syringic acid, Naringin, Quercetin, Apigenin, Genistein		
		Shoot		ABTS	IC_50_: 238.27 ± 1.61 μg/ml			Gallic acid, Syringic acid, ferulic acid, Naringin, Quercetin, Kaempferol, Genistein		
	*Galanthus transcauca-sicus* Fomin	Bulb	Methanol extract	FRAP	IC_50_: 151.21 ± 1.28 μg/ml	Vitamin C (96.15 ± 1.37) μg/ml, Vitamin E (66.84 ± 1.72 μg/ml), BHT (83.75 ± 1.87 μg/ml)	Reducing ferric ion (^3+^) to form ferrous ion (^2+^).	Gallic acid, Syringic acid, catecin, Ferulic acid, Naringin, Kaempferol, Rutin		
		Flower		FRAP	IC_50_: 137.05 ± 1.36 μg/ml			Gallic acid, Resorcinol, Syringic acid, Naringin, Quercetin, Apigenin, Genistein		
		Shoot		FRAP	IC_50_: 107.42 ± 1.03 μg/ml			Gallic acid, Syringic acid, Ferulic acid, Naringin, Quercetin, Kaempferol, Genistein		
	*Galanthus woronowii* Losinsk.	—	Hexane extract	DPPH	IC_50_: 69.07 ± 0.42 μg/ml	BHT (9.92 ± 0.23 μg/ml), BHA (5.37 ± 0.21 μg/ml), Trolox (5.77 ± 0.12 μg/ml)	Direct inhibition of ROS.	–		Genç et al. (2019)
			Chloroform extract	DPPH	IC_50_: 34.63 ± 0.21 μg/ml					
			Ethyl acetate extract	DPPH	IC_50_: 28.14 ± 0.40 μg/ml					
	*Galanthus woronowii* Losinsk.	—	Hexane extract	CUPRAC	0.49 ± 0.03 μmol TE/mg	BHT (3.63 ± 0.18), BHA (2.87 ± 0.18)	Reducing copper (2+) to copper (+).	—		
			Chloroform extract	CUPRAC	0.98 ± 0.17 μmol TE/mg					
			Ethyl acetate extract	CUPRAC	0.72 ± 0.01 μmol TE/mg					
	*Galanthus woronowii* Losinsk.	—	Hexane extract	ABTS	IC_50_: 28.51 ± 1.27 μg/ml	BHT (5.38 ± 0.18 μg/ml), BHA (8.80 ± 0.06 μg/ml), Trolox (5.57 ± 0.09 μg/ml)	Direct inhibition of cation ROS.	—		
			Chloroform extract	ABTS	IC_50_: 16.84 ± 0.49 μg/ml					
			Ethyl acetate extract	ABTS	IC_50_: 13.09 ± 0.20 μg/ml					
	*Galanthus krasnovii* Khokhr.	—	Dichloromethane extract	CUPRAC	1.15 µmol TE/mg	—	Reducing copper (^2+^) to copper (^+^).	—		Erenler et al. (2019)
			Ethyl acetate extract	CUPRAC	0.77 μmol TE/mg					
			Hexane extract	CUPRAC	0.75 μmol TE/mg					
	*Galanthus krasnovii* Khokhr.	—	Dichloromethane extract	ABTS	14.33 μg/ml	BHA (8.8 μg/ml)				
			Ethyl acetate extract	ABTS	14.98 μg/ml					
	*Galanthus nivalis* L.	Leaf	Methanol extract	DPPH	77%	Ascorbic acid (93%)		– Galanthamine	HPLC	Bulduk and Karafakıoğlu (2019)
		Leaf		ABTS	20 ± 0.78 μmol TE/100 g	Trolox		—		
		Bulb		ABTS	19 ± 0.80 μmol TE/100 g	Trolox				
	*Galanthus elwesii* Hook.f.	Leaf	Methanol extract	ABTS	20 ± 0.85 μmol TE/100 g					
		Bulb		ABTS	17 ± 0.78 μmol TE/100 g					
	*Galanthus woronowii* Losinsk.	Leaf		ABTS	23 ± 0.64 μmol TE/100 g					
		Bulbs		ABTS	21 ± 0.710 μmol TE/100 g					
Anticancer	*Galanthus kemulariae* Kuth. (accepted name*: Galanthus lagodechia-nus* Kem.-Nath.)	Aerial	Methanol extract	HCT-116	CC_50_: 36.4 ± 1.8 μg/ml	Galanthamine (>28.7 μg/ml), Tazettine (>33.1 μg/ml), Lycorine (0.88 μg/ml)	Signal-induced programmed cell death (apoptosis).	—		Jokhadze et al. (2007)
				HeLa	CC_50_: 58.2 ± 5.9 μg/ml					
				HL-60	CC_50_: 53.8 ± 6.4 μg/ml					
		Bulb	Methanol extract	HCT-116	CC_50_: 12.2 ± 2.7 μg/ml					
				HeLa	CC_50_: 37.1 ± 4.7 μg/ml					
				HL-60	CC_50_: 34.3 ± 3.9 μg/ml					
	*Galanthus lagodechia-nus* Kem.-Nath.	Bulb	Methanol extract	HCT-116	CC_50_: 11.1 ± 3.4 μg/ml	Galanthamine (>28.7 μg/ml), Tazettine (>33.1 μg/ml), Lycorine (0.88 μg/ml)		—		
				HeLa	CC_50_: 34.8 ± 6.3 μg/ml					
				HL-60	CC_50_: 45.6 ± 3.5 μg/ml					
	*Galanthus woronowii* Losinsk.	Aerial	Methanol extract	HCT-116	CC_50_: 22.0 ± 3.8 μg/ml	Galanthamine (>28.7 μg/ml), Tazettine (>33.1 μg/ml), Lycorine (0.88 μg/ml)		–		
				HeLa	CC_50_: 41.3 ± 3.3 μg/ml					
				HL-60	CC_50_: 39.4 ± 2.8 μg/ml					
	*Galanthus krasnovii* Khokhr.	Bulb	Methanol extract	HCT-116	CC_50_: 5.8 ± 0.9 μg/ml	Galanthamine (>28.7 μg/ml), Tazettine (>33.1 μg/ml), Lycorine (0.88 μg/ml)		—		
				HeLa	CC_50_: 15.4 ± 3.7 μg/ml					
				HL-60	CC_50_: 13.8 ± 1.2 μg/ml					
		Bulb	Methanol extract	HCT-116	CC_50_: 7.7 ± 1.6 μg/ml					
				HeLa	CC_50_: 18.9 ± 3.9 μg/ml					
				HL-60	CC_50_: 22.0 ± 2.4 μg/ml					
	*Galanthus alpinus* Sosn.	Bulb	Methanol extract	HCT-116	CC_50_: 9.6 ± 0.8 μg/ml	Galanthamine (>28.7 μg/ml), Tazettine (>33.1 μg/ml), Lycorine (0.88 μg/ml)		—		
				HeLa	CC_50_: 21.3 ± 4.5 μg/ml					
				HL-60	CC_50_: 23.7 ± 1.7 μg/ml					
	*Galanthus shaoricus* Kem.-Nath.	Bulb	Methanol extract	HCT-116	CC_50_: 8.9 ± 1.6 μg/ml	Galanthamine (>28.7 μg/ml), Tazettine (>33.1 μg/ml), Lycorine (0.88 μg/ml)		—		
				HeLa	CC_50_: 17.2 ± 2.1 μg/ml					
				HL-60	CC_50_: 16.4 ± 0.9 μg/ml					
	*Galanthus platyphyllu-s* Traub and Moldenke	Bulb	Methanol extract	HCT-116	CC_50_: 14.2 ± 2.7 μg/ml	alanthamine (>28.7 μg/ml), Tazettine (>33.1 μg/ml), Lycorine (0.88 μg/ml)		—		
				HeLa	CC_50_: 11.5 ± 1.7 μg/ml					
				HL-60	CC_50_: 19.1 ± 1.0 μg/ml					
	*Galanthus caucasicus* (Baker) Grossh. (accepted name: *Galanthus alpinus* var. *alpinus*)	Aerial	Methanol extract	HCT-116	CC_50_: 49.5 ± 4.8 μg/ml	Galanthamine (>28.7 μg/ml), Tazettine (>33.1 μg/ml), Lycorine (0.88 μg/ml)		—		
				HeLa	CC_50_: 42.8 ± 2.8 μg/ml					
				HL-60	CC_50_: 39.3 ± 2.3 μg/ml					
		Bulb	Methanol extract	HCT-116	CC_50_: 23.4 ± 3.7 μg/ml	Galanthamine (>28.7 μg/ml), Tazettine (>33.1 μg/ml), Lycorine (0.88 μg/ml)				
				HeLa	CC_50_: 32.1 ± 3.7 μg/ml					
				HL-60	CC_50_: 31.9 ± 1.5 μg/ml					

^a^ Effective dose: Dose that gives significant results with p < 0.05, p < 0.01, p < 0.001.

^1^H-NMR, hydrogen-1 nuclear magnetic resonance; ABTS, 2,2′-azino-bis(3-ethylbenzothiazoline-6-sulfonic acid); ACh, acetylcholine; AChE, acetylcholinesterase; BHA, butylated hydroxyanisole; BHT, butylated hydroxytoluene; CC_50_, half maximal cytotoxic and inhibitory concentration; DPPH, 2,2-diphenyl-1-picrylhydrazyl; EC_50_, half maximal effective concentration; EIMS, electron ionization mass spectrometry; GC-MS, gas chromatography-mass spectrometry; HPLC, high performance liquid chromatography, IC_50_, half maximal inhibitory concentration; MIC, minimal inhibitory concentration; MFC, minimal fungicidal concentration; NA, no activity; NMR, nuclear magnetic resonance; ROS, reactive oxygen species; SE, standard error; TLC, thin layer chromatography.

### Anticholinesterase Activity

Acetylcholinesterase (AChE), aenzyme remain a highly viable target to alleviate the symptoms of Alzheimer’s disease (AD) (Kostelník and Pohanka, 2018). AChE (specific cholinsterase) is present in nervous system and terminates neurotransmission, while the activity of BChE is increase during the late stage of AD (Mesulam and Geula, 1994; Khaw et al., 2014; Kostelník and Pohanka, 2018). Galanthamine is known to enhance the activity of acetylcholine (ACh) by inhibiting the enzyme AChE and functions as a nicotinic activator by interacting with nicotinic ACh receptors (nAChRs) in the brain (Maelicke et al., 1997). The interaction between the Ach inhibitor and nAChR induces conformational change of the receptor molecule, and subsequent activation of nAChRs is believed to have protective effects against β-amyloid cytotoxicity of neuron cells (Coyle and Kershaw, 2001). Snowdrops are important source of anti-neurodegeneration compound “galanthamine” thanks to the traditional knowledge in which the extract has been used in folk medicine for neurological conditions (Ago et al., 2011). Due to limited number of drugs available for the management of Alzheimer disease, significant efforts have been made to explore anticholinesterase inhibitor from medicinal plants (Khaw et al., 2014; Tan et al., 2014; Jamila et al., 2015; Liew et al., 2015; Khaw et al., 2020).

The anti-cholinesterase activities of the *Galanthus* spp including *Galanthus Nivalis*, *Galanthus elwesii*, *Galanthus ikariae*, *Galanthus gracilis*, *Galanthus xvalentinen*, *Galanthus rizehensis*, *Galanthus cilicicus*, were assessed *in-vitro* by determining their inhibitory activities via Ellman method (Table 2). Rhee et al. (2003) showed that the methanol extract of *G. nivalis* had 96% inhibition against AChE (Rhee et al., 2003). Chloroform:methanol (1 : 1) extracts of the bulbs of *G. elwesii* and *G. ikariae* inhibited AChE at 73.18 and 75.56% (10 μg/ml), comparable to the alkaloid extracts at 77.23 and 76.96% (10 μg/ml) (Orhan and Şener, 2005). Phytochemical study of alkaloid extract of *G. ikariae* yielded amaryllidaceae-type alkaloids, including lycorine (IC_50_ = 3.16 µM), tazettine, crinine, galanthamine (IC_50_ = 3.2 µM), 3-epi-hydroxybulbispermine and 2-demethoxymontanine. A study of Kaya and colleagues demonstrated that bulb and aeries parts of *G. cilicicus* selective towards AChE than BuChE, suggesting the present of selective AChE compounds within the extract.

Similarly, methanol extracts of the bulb and aerial part of *G. elwesii* were selectively inhibited AChE (Bozkurt et al., 2013a; Kaya et al., 2017). Subsequent GCMS analysis revealed the present of alkaloids in the *G. elwesii* extract including Galanthamine, O-methylleucotamine, hordenine and sanguinine (Bozkurt et al., 2017). The alkaloid extracts of the *G. gracilis* bulb and *G. xvalentinei* nothosubsp. Subplicatus were moderately inhibiting AChE with the IC_50_ of 11.82–25.5 µg/ml (Sarikaya et al., 2013; Bozkurt-Sarikaya et al., 2014). The bulb of *G. krasnovii* alkaloid was dual cholinesterase inhibitor with the IC_50_ of 8.26 µg/ml (AChE) and IC_50_ of 6.23 µg/ml (BuChE) (Bozkurt et al., 2020). GCMS analysis revealed that anhydrolycorine and 11,12-didehydroanhydrolycorine were the dominant compounds in the extract contribute to the inhibitory activities.

The findings showed that alkaloids from *Galanthus* spp played an important role in cholinesterase inhibitory activities. Among the alkaloids, lycorine-type alkaloids dominated in the studied extracts. Galanthamine and tazettine-type alkaloids were present in very low amounts. The alkaloid content in the bulb was more prominent than the aerial parts. The findings showed that inhibitory activity might be due to the synergistic interactions between the alkaloids within the extract. Taking into account that existing drugs are effective mild to moderate progression of AD and presenting considerable side effects, the search for effective and selective cholinesterase inhibitors with minimum side effects is imperative. It can be conclude that, the bulb of *Galanthus* spp. can be served as a source of anticholinesterase alkaloids in addition to their ornamental properties.

### Antimicrobial Activity

The emergence of new infectious diseases and drug resistance to antibiotic is one of the biggest threats to global health (Ventola, 2015). Antimicrobial, including antibacterial, antifungal, antiviral and antiprotozoal agents are becoming ineffective, attributed to the overuse and misuse of current existing drugs which leads to resistance (Interagency Coordination Group, 2019). On top of that, diminishing antibiotic pipeline resulted in lesser treatment options against multiple drug resistance pathogens and responsible for at least 700,000 casualties each year (Interagency Coordination Group, 2019). Natural products are promising new drug candidates in treating antibiotic-resistant infections. Natural products have evolved in natural selection process adapting to various abiotic and biotic stresses where abundant of undiscovered biologically active metabolites for drug discovery. Natural products have always been an important part of drug discovery and intense research has been conducted in this area since the discovery of penicillin in the forties.

#### Antibacterial

Turker and Koyluoglu (2012) reported antibacterial activity of ethanol extract of *G. Plicatus* against Gram-positive *Staphylococcus epidermidis* and *Staphylococcus pyrogenes* and Gram-negative *Proteus vulgaris* and *Klebsiella pneumoniae* obtained from disc-diffusion method (Turker and Koyluoglu, 2012). Growth inhibitions (7.25 ± 0.25 to 12.50 ± 0.50 mm) were compared with positive controls such as chloramphenicol, tetracycline, ampicillin, carbenicillin and erythromycin. In another study, the ethanol and chloroform extracts of *G. transcaucasicus* showed antibacterial activity against *Bacillus subtilis* and *Staphylococcus aureus* at MIC values of 9.275 mg/ml and 1.17 mg/ml, respectively (Sharifzadeh et al., 2010). The methanol extracts of the bulb, flower and shoot of *G. transcaucasicus* were evaluated for their antibacterial activity against *Bacillus subtilis*, *Bacillus cereus*, *Staphylococcus aureus*, *Escherichia coli* and *Pseudomonas aeruginosa* (Karimi et al., 2018). Overall, the antibacterial activity of shoot extract appeared to be most potent followed by flower and bulb extracts. The main and predominant volatile compounds such as acetic acid (13.6%), 2,3-Butanediol (43.13%) and 2-Furancarboxaldehyde (68.77%) were major in shoot, flower and bulb extracts of *G. transcaucasicus*, respectively. *G. nivalis* extract has demonstrated moderate anti-staphylococcal activity, with the minimal inhibitory concentration (MIC) value of 19.53 μg/ml (Benedec et al., 2018). Interestingly, *G. nivalis* extract exhibited comparable antibacterial activity with standard drug, gentamicin. Phytochemical analysis of G. *nivalis* extract revealed that chlorogenic acid (2976.19 ± 12.80 μg/g) was the main constituent, followed by *p*-coumaric acid (73.02 ± 0.07 μg/g), ferulic acid (26.80 ± 0.19 μg/g), isoquercitrin (25.08 ± 0.31 μg/g) and quercitrin (11.13 ± 0.06 μg/g).

#### Antifungal

The antifungal activity of ethanol extract of the bulb of *G. transcaucasicus* against yeast *Candida albicans* stood at MIC values of 19.53 μg/ml to 2,500 μg/ml (Sharifzadeh et al., 2010). A study by Ločárek and colleagues showed that alkaloid extract of the bulb of *G. elwesii* inhibited the growth of *Candida* spp. and *Lodderomyces elongisporus* (Ločárek et al., 2015). Galanthamine was the major compound in the alkaloid extract, followed by tazettine and minute amount of haemantamine as analyzed by GCMS. Benedec et al. (2018) reported antifungal activity of *G. nivalis* against *C. albicans* and filamentous fungi, *Aspergillus brasiliensis* (Benedec et al., 2018). Phytochemical analysis showed that chlorogenic acid was the dominant phenolic acid within *G. nivalis* extract.

#### Antiprotozoal

Amaryllidaceae alkaloids have previously been tested to possess antiparasitic activities (Campbell et al., 2000; Toriizuka et al., 2008) Antiprotozoal activity of the compounds isolated from alkaloid extract was tested against a panel of parasitic protozoa consisting of *Trypanosoma brucei rhodesiense, Trypanosoma cruzi*, *Leishmania donovani,* and *Plasmodium falciparum*, which are responsible for human African trypanosomiasis (sleeping sickness), American trypanosomiasis, Kalaazar (visceral leishmaniasis) and malaria were evaluated *in vitro* by Plasmodial FAS-II enzyme inhibition assay (Kaya et al., 2011). Arolycoricidine (+)-haemanthamine, dihydrolycorine, and protopine were active against *T. b. rhodesiense*, while (+)-haemanthamine was active against *T. cruzi* with the IC_50_ less than 10 μg/ml. Arolycoricidine (+)-haemanthamine, stylopine and protopine were reported potentially against *P. falciparum, where* stylopine and protopine exhibited sub-microgram inhibition with the IC_50_ values of 0.23 and 0.50 μg/ml In addition, stylopine and protopine demonstrated good cytotoxicity (L6 and KB cells) selectivity index grant these compounds as promising lead for further development. The study showed that most of the active compounds are of lycorine type-alkaloids, in which O-methylnorbelladine (–)-dihydrolycorine and (+)-8-O-demethylmaritidine are being reported here for the first time from the genus *Galanthus*. Amaryllidaceae-derived haemanthamine displayed remarkable cytotoxicity against primary mammalian cell line (L6) and the human carcinoma cell line (KB) (Kaya et al., 2011).

Lycorine, an Amaryllidaceae alkaloid from snowdrop possesses strong antimalarial activity (Khalifa et al., 2018). It was potently inhibited the growth of *P. falciparum*, the causative agent of malaria, with a low cytotoxic profile against human hepatocarcinoma cells (HepG2) (Gonring-Salarini et al., 2019).

In general, antimalarial agents manifest their action by targeting enzymes associated with the plasmodial FAS-II biosynthetic pathways (Nair and Staden, 2019). It inhibits DNA topoisomerase-I activity which is required for cell growth in parasites and causes cell cycle arrest *in vivo* (Cortese et al., 1983). The results suggested that the antimalarial activities of lycorine derivatives might be due to the free hydroxyl groups at C-1 and C-2 or esterified as acetates or isobutyrates. The presence of a double bond between C-2 and C-3 is important for the activity (Cedrón et al., 2010; He et al., 2015). Overall, these results suggested that *Galanthus* spp. is potentialantiprotozoal agent for further development.

#### Antiviral

Among the microbes, virus infection has emerged as a leading cause of morbidity and mortality worldwide (Luo and Gao, 2020). Recent outbreak has underscored their prevention as a critical issue in safeguarding public health with very limited number of antivirals drugs, vaccines and antiviral therapies available (Babar et al., 2013).

Lectin from snowdrops is being investigated for its anti-viral potential. The *Galanthus nivalis* agglutinin (GNA) was identified and purified from the bulb of snowdrop (Van Damme et al., 1987). GNA is known to possess virucidal properties against human immunodeficiency virus (HIV) at the EC_50_ = 0.12 ± 0.07 μg/ml to 4.7 ± 3 μg/ml (Balzarini et al., 2004). The molecular mechanisms of GNA exerting antiviral activities via carbohydrate-binding activities, thereby blocking the entry of the virus into its target cells and transmission of the virus by deleting the glycan shield in its envelope protein, thus neutralizing antibody.

*G. elwesii*’s ethanol extract was tested for its anti-herpes simplex virus (HSV) and anti-sindbis virus (SINV) activity. *G. elwesii* has higher activity in the virucidal (8 µg/ml) assay than the plaque-forming assay (24 µg/ml) (Hudson et al., 2000). *G. elwesii* extract was potent against SINV, it showed anti-SINV activity at the dose of 16 µg/ml.

Most of the mannose-binding lectins exert anti-coronavirus potential except the lectins from garlic (Keyaerts et al., 2007). They interfered with viral attachment in early stage of replication cycle and suppressed the growth by interacting at the end of the infectious virus cycle. The virucidal effect of GNA against SARS-CoV was recorded at EC_50_ of 6.2 ± 0.6 μg/ml (Keyaerts et al., 2007). Other GNA-related lectins may exert anti-influenza activities by competitively blocking the combination of influenza A virus envelope glycoprotein haemagglutinin (HA) with its corresponding sialic acid-linked receptor in the host cell, such as H1N1 (Yang et al., 2013). A study evaluated the antiviral potential of plant lectins from a collection of medicinal plants on feline infectious peritonitis virus (FIPV) infected cells. The results indicated that plants derived mannose-binding lections had strongest anti-coronavirus activitity and *Galanthus nivalis* was one of the coronavirus-inhibiting plants (Adams, 2020).

To sum up, lectin GNA might be a potential target for further development for its anti-CoV potential. Although no CoV treatments have been approved, pharmacotherapies for MERS-CoV and SARS-CoV may lay the foundation for treatment of the novel human Coronavirus Disease 2019 (COVID-19).

### Antioxidant Activity

Natural antioxidants play a role in preventing cellular free radicals or reactive oxygen species (ROS) formation as well as facilitating repair process from the damage caused by ROS induced oxidative stress which involves in various chronic diseases, such as atherosclerosis, myocardial infections, cancer and neurodegenerative diseases (Bulduk and Karafakıoğlu, 2019). Antioxidants can act as chain breakers, radical scavengers, singlet oxygen quenchers, hydroperoxides decomposers, and pro-oxidative metal ions chelators (Pisoschi et al., 2016).

The antioxidant potential of the aerial and bulb of *G. reginae-olgae* was determined by free radical scavenging DPPH, lipid peroxidation and β-carotene bleaching tests (Conforti et al., 2010). The result showed that methanol extracts of aerial and bulb of *G. reginae-olgae* had moderate DPPH scavenging potential. Further fractionation of the extracts indicate that the strongest DPPH scavenging of aerial part was ethyl acetate fraction, while alkaloid fraction of bulb showed highest scavenging potential. The results showed that the DPPH scavenging activity of ethyl acetate and alkaloid fractions of aerial and bulb attributed to their distinct chemical diversity The shoot of G. *transcaucasicus* exhibited higher antioxidant activities compare to bulb and flower that concurred with the high phenolic and flavonoid compounds in shoot. In a comparative study, the ethanol extract of *G. woronowii* exhibited highest DPPH and 2,2′-azino-bis(3-ethylbenzothiazoline-6-sulphonic acid (ABTS) scavenging activity (IC_50_ = 28.14 μg/ml and 13.09 μg/ml, respectively) (Genç et al., 2019). While dichloromethane extract displayed greater reducing potential in cupric ion reducing power assay that ethanol extract. Antioxidant activity of hexane, dichloromethane and ethyl acetate extracts of *G. krasnovii* were investigated via DPPH and ABTS radical scavenging and cupric ion reducing power assay (Erenler et al., 2019). Dichloromethane extract demonstrated the highest ABTS activity (IC_50_ = 14.33 μg/ml) and reducing power (1.15 µmol TE/mg). DPPH and ABTS method were also been used to investigate the methanol extracts of the leaf and bulb of three *Galanthus* spp. (Bulduk and Karafakıoğlu, 2019). The *G. woronowii* leaf extract recorded the highest DPPH scavenging activity (77%), whereas all extracts from *G. nivalis*, *G. elwesii* and *G. woronowii* showed comparable ABTS scavenging activity (17 ± 0.78 – 23 ± 0.64 µmol TE/100 g). HPLC analysis showed that content of galantamine was higher in the aerial parts (leaves) when compared to the underground parts (bulbs) which may contributed to the higher scavenging activity of the leaf extract.

Apparently, *Galanthus* spp. appears to be potent source of antioxidants which are enriched with various phytochemicals phenolic acids, flavonoids, and alkaloids (Karimi et al., 2018). It is envisaged that secondary metabolites from *Galanthus* spp. may reduce the risk and slow down the progression of chronic diseases including cancers, cardiovascular diseases and neurodegenerative diseases.

### Anticancer Activity

Cancer is a chronic disease, which is account for millions of deaths each year (Tan et al., 2016; Tay et al., 2019). Chemotherapy, radiotherapy and recently, immunotherapy are essential means for the treatment of cancers. Severe toxicity and cell resistance to drugs are the major drawback in conventional cancer therapies. In order to circumvent these issues, new cellular targets and anticancer agents are needed, especially those of natural origin. From 1981 to 2002, natural products were the basis of 74% of all new chemical entities for cancer (Demain and Vaishnav, 2011).

Eight different *Galanthus* species were tested for their anticancer activity on Human colorectal carcinoma cells (HCT-116), Human promyelocytic leukemia cells (Hela) and Human cervical cancer cells (HL-60) (Jokhadze et al., 2007). All methanol extracts from the galanthus species showed cytotoxic activities, in which the bulbs had higher activity than the aerial parts. Majority of the species were more active against HCT-116 cells, except G. *platyphyllus* bulbs were more active against HeLa cells than other cell lines, indicating an interesting specificity that should be investigated in future studies. The bulbs of G. *woronowii*, G. *krasnowii*, G. *shaoricus* and G. *alpinus* were the most cytotoxic (IC_50_ < 10 µg/ml) on HCT-116 cells. Lycorine had cytotoxicity against HCT-116, HL-60 and Hela cells with IC_50_ of 3.1, 8.2, and 9.3 µM. Meanwhile, galanthamine and tazettine were weakly cytotoxic against HCT-116, HL-60 and Hela cells, with IC_50_ > 100 µM. It is suggesting that the present of lycorine in the *Galanthus s*pp contributed to the cytotoxic effects on the tested cancer cells. The search for novel anticancer agents from natural sources has been successful worldwide. For over 50 years, natural products have served us well in combating cancer and is still a priority goal for cancer therapy, due to the chemotherapeutic drugs resistance.

## Conclusion and Future Perspectives

Natural products remain to be a wealthy source for the identification of novel therapeutic agents for the treatment of human diseases. Plants contain a significant numbers of phytochemical components, most of which are known to be biologically active and responsible for various pharmacological activities. It was demonstrated that plant secondary metabolites are preferred natural antioxidants than synthetic ones due to safety concerns. Given the natural abundance of bioactive compounds in this plant, *Galanthus* spp. can be recognized as an interesting source of natural products with a wide range of biological activities. This review highlights the importance of bioactive substances of various extracts of *Galanthus* spp. on anti-cholinesterase inhibitory activity and other diseases, supporting the therapeutic possibilities for the use of snowdrops. The most promising compound is galanthamine which exhibited greater activity than tazettine, crinine and lycorine. However, current research on the underlying mechanism of actions and the exact chemical constituent involved are scarce. Apart from the above mentioned activities, other ethnopharmacological uses of snowdrops need to be substantiated with strong scientific studies for its extensive usage in various therapies. Thus, this review may serve as a guide for future researchers in pharmacology to conduct further studies on these plants by providing different perspective. The discussion is expected to inspire further isolation, identification, mechanism of actions and synthetic studies of the existing and novel active compounds from the *Galanthus* spp. to gain a better understanding of the basis of the activity at the cellular and molecular level in future.
